# Approaches for Controlling Antibody-Mediated Allograft Rejection Through Targeting B Cells

**DOI:** 10.3389/fimmu.2021.682334

**Published:** 2021-07-01

**Authors:** Yoshiko Matsuda, Takeshi Watanabe, Xiao-Kang Li

**Affiliations:** ^1^ Division of Transplantation Immunology, National Research Institute for Child Health and Development, Tokyo, Japan; ^2^ Laboratory of Immunology, Institute for Frontier Life and Medical Sciences, Kyoto University, Kyoto, Japan

**Keywords:** antibody-mediated allograft rejection, naïve-B cell, memory-B cell, germinal center B cell, long-lived plasma cell, B cell biology

## Abstract

Both acute and chronic antibody-mediated allograft rejection (AMR), which are directly mediated by B cells, remain difficult to treat. Long-lived plasma cells (LLPCs) in bone marrow (BM) play a crucial role in the production of the antibodies that induce AMR. However, LLPCs survive through a T cell-independent mechanism and resist conventional immunosuppressive therapy. Desensitization therapy is therefore performed, although it is accompanied by severe side effects and the pathological condition may be at an irreversible stage when these antibodies, which induce AMR development, are detected in the serum. In other words, AMR control requires the development of a diagnostic method that predicts its onset before LLPC differentiation and enables therapeutic intervention and the establishment of humoral immune monitoring methods providing more detailed information, including individual differences in the susceptibility to immunosuppressive agents and the pathological conditions. In this study, we reviewed recent studies related to the direct or indirect involvement of immunocompetent cells in the differentiation of naïve-B cells into LLPCs, the limitations of conventional methods, and the possible development of novel control methods in the context of AMR. This information will significantly contribute to the development of clinical applications for AMR and improve the prognosis of patients who undergo organ transplantation.

## Introduction

Experiments by using thymectomized mice or chickens conducted during the mid-1960s show that T cell mediated immunity in tissue and organ allografts. Consequently, most immunosuppressive therapies for preventing allograft rejection were targeted on T cells, and the studies of the *in vitro* effects of immunosuppressive agents against the proliferating T cells significantly contributed to controlling T cell-mediated rejection (TCMR) (1). Although T cells mediate the activation of the humoral immune response to transplanted grafts through activating B cells, efforts to control antibody-mediated allograft rejection (AMR) using conventional immunosuppressive therapy have been still challenging until now (2–16).

Therefore, further understanding of B cell biology related to the differentiation of long-lived plasma cells (LLPCs) and the regulation of the production of antibodies that induce the development of AMR are required to improve disease prognosis.

During AMR development, naïve-B cells recognize donor-specific human leukocyte antigen (HLA) and differentiate into activated B cells. These activated B cells undergo negative selection in germinal centers (GCs), and only cells with high affinity for donor-specific HLA survive, subsequently differentiate into memory B cells (MBCs) or migrate into the bone marrow (BM), and differentiate into LLPCs. LLPCs maintain long-term donor-specific HLA antibodies (DSAs) production (17–19) and conventional immunosuppressive therapy is ineffective for removing these PCs, because their survival is independent on the activities of T cells and expression of CD20 is reduced on these PCs (19, 20). Moreover, the intramedullary environment may prevent these PCs from undergoing apoptosis induced by desensitization therapy (21). Therefore, the development of accurate and rapid techniques to evaluate humoral immune activation targeting transplanted grafts, independent of antibodies, are urgently required to control AMR.

Identifying of the antigen specificity of MBCs circulating in the peripheral blood, as measured using an ELISpot assay or *in vitro* assay system, will be useful to evaluate the activation of the humoral immune response to donor-specific HLA antigens (22–27). Furthermore, microarray techniques and next-generation sequencing (NGS) may provide detailed information that will contribute to therapeutic control of AMR, including the identification of antibodies that injure transplants and individual differences in patients’ susceptibilities to immunosuppressive agents (28–36).

In addition, the components of humoral immunity involved in immune tolerance as well as in the repair of injured tissues are attracting attention, particularly in the fields of autoimmune diseases, severe infectious diseases, and others (37–42). Here, we discuss the possibility of AMR control by referring to the involvement mechanism of these components in the humoral immunity-associated pathology and the possibilities of these components as well as novel humoral immune monitoring in solving the problems associated with conventional AMR control.

## Molecular Pathophysiology in AMR

**Figure 1:**
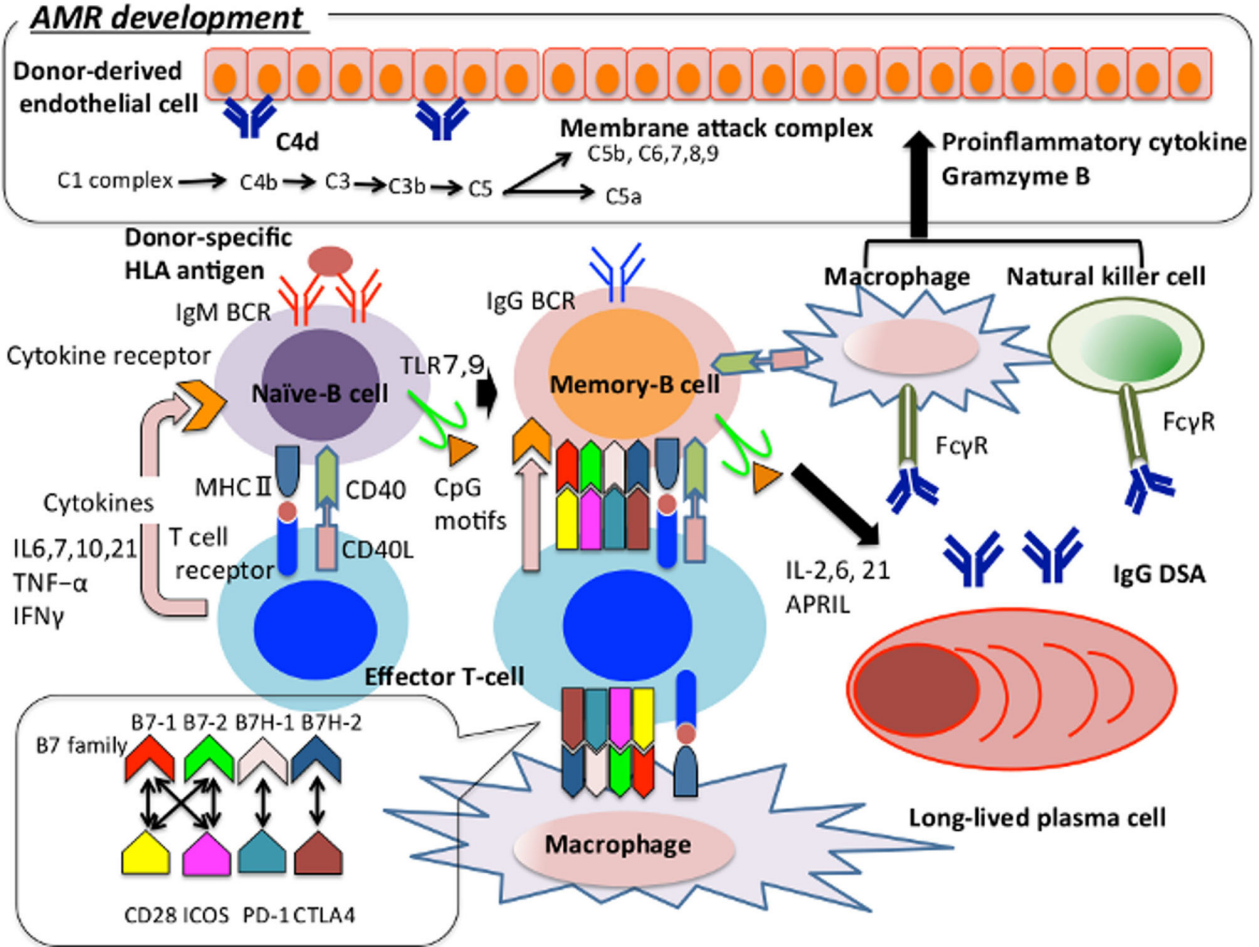
The pathway of naïve-B cell differentiation into plasma cell in the context of AMR development. We show how immunocompetent cells are involved in the onset of AMR, focusing on B cells. Naïve-B cell recognises donor-specific HLA antigen and present the antigen peptides from MHCII to T cell receptor and activate T cells. The activated T cells regulate the growth and survival of B cells through the production of IL4, 6, 7, 10, 21, TNF-α, IFNγ, etc (46). Activated B cell migrates to secondary lymphoid tissue, class-switches to IgG and then undergoes gene conversion and hypersomatic mutation in the germinal center to have a high affinity for donor-specific HLA antigen. These cells migrate into the bone marrow and differentiate into long-lived plasma cells, and keep producing IgG DSAs for a long term. Furthermore, TLR 7/9 is expressed on the surface of naïve-B cells. TLR7 plays an important role in T cell activation and germinal center B-cell development (24). TLR9 recognizes the CpG motif and supports the survival and proliferation of B cells through IL-6 production and T cell activation (47). T cells regulate B cells’ activation and sustain the interaction between follicular helper T cells and germinal center B cells through the association between the B7 family and CD28, ICOS, PD-1, CTLA-4 expressed by B cells, and CD 40 ligand and CD40 expressed by B cells. Regarding macrophages and natural killer cells function as antigen-presenting cells and induce B cells and T cells activation. IgG-type DSA binds to Fcγ on the macrophages and natural killer cells’ surface and activates them. Complement activates adaptive immunity against thymus dependent antigen, or it activates the membrane attack complex and induces inflammation and tissue damage on vascular endothelial cells, thereby supporting the onset of AMR. AMR, Antibody-mediated allograft rejection; APRIL, A proliferation-inducing ligand; BCR, B cell receptor; CD40L, CD40 ligand; CTLA-4, Cytotoxic T-lymphocyte associated antigen 4; DSA, Donor-specific HLA antibody; FcγR, Fc gamma receptor; IFN, Interferon; ICOS, Inducible T-cell co-stimulator; MHC, Major histocompatibility complex; PD-1, Programmed cell death – 1; TNF, Tumor Necrosis Factor; TLR, Tool-like receptor.

In the pathways or events leading to the development AMR, molecules such as donor-specific HLA antigens, non-HLA antigens, and self-antigens are recognized by antigen-presenting cells (APCs) that express the major histocompatibility complex (MHC) II on their surface. These latter molecules are presented to follicular helper T cells (Tfhs) through the interaction of MHC II with the T cell receptor (TCR), resulting in the activation of Tfhs (17). The B cell receptor (BCR), which is expressed on the surface of naïve-B cells, is activated through cross-linking to the aforementioned antigens in a T cell-dependent manner, and B cells monoclonally proliferate after stimulation by activated Tfhs in secondary lymphoid tissues. These activated B cells form GCs. After immunoglobulin class switching, they undergo affinity maturation and the cells with low affinity for foreign antigens or high affinity for self-antigens undergo apoptosis as a negative selection mechanism, followed by differentiation into MBCs or LLPCs that bind these antigens with high affinity. MBCs migrate to secondary lymphoid tissue to counter the ensuing invasion by foreign antigens, and LLPCs persist and continue to produce antibodies that induce AMR (17, 18, 43–45) (**Figure 1**).

Regarding the involvement of T cell in AMR development, Tfhs reside in the peripheral secondary lymphoid tissues and strongly promote the production of antigen-specific antibodies through the support of the GC reactions. The B7 (CD80/86) family of costimulatory molecules B7-1 and B7-2 binds to CD28 and cytotoxic T-lymphocyte associated antigen 4 (CTLA-4) on the surface of T cells binds more strongly to CD80/86 than to CD28 to release control over the generation of Tfhs (2). Human B7 homolog 1 (B7H-1) binds programmed death-ligand 1 (PD-L1) expressed by activated T cells and inhibits T cell activation through the interaction between PD-L1 and programmed cell death1 (PD-1). Human B7 homolog 2 (B7H-2) binds inducible T cell costimulator (ICOS) expressed by CD4/CD8 T cells to increase the secretion of interferon (IFN)-γ and interleukin (IL)-10. ICOS-ligand is expressed on the surface of B cells and supports their migration into B follicles (3, 4). In addition, Thfs support the formation of the GC through the interaction between CD40 and CD40-ligand (CD40-L) (5–9); and the G-protein-coupled receptor sphingosine-1-phosphate receptor 2 (S1PR2) cooperates with CXCR5 to retain Thfs in the GC (10). Members of the signaling lymphocyte activation molecule (SLAM) family act to maintain stable contacts between Tfhs and GC B cells to maintain the differentiation of Thfs, formation of the GC, and proliferation and survival of LLPCs and MBCs (11–16). In contrast, follicular regulatory T cells (Tfrs) inhibit the production of IL-21 by Thfs, and the CTLA-4-mediated signals cooperate with IL-10 and transforming growth factor (TGF)-beta to support the inhibitory effect on humoral immunity (48).

Macrophages (MPs) and natural killer (NK) cells also play an important role in the development of AMR (49). MPs present antigens to Tfhs *via* MHC class II, serve as a source of co-stimulatory signaling and cytokines required for activation of T cells, and subsequently activate B cells (50). Moreover, IgG DSAs have been revealed to bind to Fc gamma receptor (FcγR) such as FcγRIIIa on the cell membrane of MPs and NK cells, and subsequently, these cells are activated. These activated cells produce proinflammatory cytokines such as IFN-γ, Tumor Necrosis Factor (TNF), and granzyme B, which induce coagulation, inflammation, vascular permeability, and leukocyte trafficking on the vascular endothelium (51).

Regarding the involvement of the complement pathway in AMR development, the humoral immune response to thymus-dependent (TD) antigens requires complementary activation, which is required for the localization of the antigen and C3 ligand to follicular dendritic cells (FDCs). These events maintain the long-term memory function of B cells (52).

As the detailed involvement mechanism in B cell activation and proliferation, the interaction of C3 fragments with CD21 is required for the internalization of antigens by B cell and their presentation. CD21 is a receptor that binds to complement C3d and Epstein–Barr virus and is expressed by mature B cells and FDCs. CD-21 plays a crucial role in B cell activation (53). The internalized antigen is presented to T cells by MHCs II and I, and it then activates T cells (54–57). Therefore, complement activation is required for the uptake of antigen by B cell and inhibiting CD21-mediated signal with polyclonal antibodies significantly inhibits antigen uptake and presentation. Furthermore, B cell uptake of C3d-coated antigen and engagement of the BCR and CD21 by C3d-opsonized antigen is required for the formation and maintenance of the GC and differentiation of MBCs into PCs (57, 58). CD35, an antagonist of CD21, inhibits B cell activation (59), and the CD21/CD19/Target of the Antiproliferative Antibody-1 (Tapa-1) receptor reduces the threshold of survival of follicular B cells and serves as a unique signal that promotes their survival in the GCs, which, in turn, contributes to adaptive immunity (52).

Therefore, complement activation indirectly induces the development of AMR through B cell activation or directly induces inflammation and damage in vascular endothelial cells through formation of the membrane attack complex (60).

## Challenges of Conventional AMR Control

Although the pathway described in “MOLECULAR PATHOPHYSIOLOGY IN AMR” is inhibited by immunosuppressive therapy, AMR control remains challenging.

The cause is that signals from immunocompetent cells, including T cells, to B cells are not well regulated, and humoral immune responses to transplanted graft have not been accurately detailed.

Furthermore, B cell subsets include IL-10-producing regulatory B cells (Bregs) (CD19^+^CD24^high^CD38^high^ transitional immature B cells), whose reduction is associated with the incidence of posttransplant rejection (61, 62). Regulatory T cells (Tregs) induce tolerance to transplanted grafts through suppression of the humoral immune response. Calcineurin inhibitor (CNI) and mammalian Target of Rapamycin (mTOR) reduce the levels of Bregs, whereas mTOR expands and CNI reduces the levels of Tregs (63). Under pathological conditions, common variable immunodeficiency (CVID) is a pathology characterized by the loss of resistance to bacterial and viral infections due to decreased antibody production. This disease is associated with decreased expression of a proliferation-inducing ligand (APRIL), which supports the growth and survival of MBCs and PCs. An anti-APRIL antibody may serve as a therapeutic agent for SLE (64, 65). In contrast, APRIL supports the growth and survival of naïve-B cells *in vitro*, and administration of an anti-APRIL antibody may therefore suppress immune tolerance mediated by naïve-B cells (66).

In the other words, some immunosuppressive agents may inhibit the normal functions of these regulatory cells and the identification of the mechanisms through which the populations of these regulatory cells are reduced by immunosuppressive therapy and the differences of drug susceptibilities in subset-specific lymphocyte may help development of more appropriated immunosuppressive agents that maintain the functions of these regulatory cells. For example, IL-2 lengthens the survival and enhances the suppressive effects of Tregs, which increases the survival rates of transplanted grafts, because the decrease in the number of Tregs by CNI is the result of limiting the activity of IL-2 (67).

In addition, desensitization therapies such as plasmapheresis and low-dose intravenous immunoglobulin (IVig), alone or combined with recombinant antithymocyte globulin (rATG), do not significantly influence the number of CD138^+^ antibody-secreting cells (ASCs), alloantibody production, and the frequency of HLA-producing ASCs in the BM before and after treatment (68). The resistance of ASCs to IVig and rATG may be explained by the inhibition of apoptosis mediated by the microenvironment of the BM and soluble and factors produced from the BM niche (21). Alternatively, the interaction of some receptors expressed on LLPC and their ligands expressed on niche play a critical role in the migration of LLPCs to the BM and their longevity in the BM (20, 69, 70). In the other words, the removal of antibody from the serum using desensitization therapy may not be mediated by the reduction of the numbers and functions of ASCs, but by antibody adsorption to the transplanted graft (68). Thus, further elucidation of the mechanisms of drug resistance in LLPCs and blockade of the signals supporting the migration of LLPCs into the BM and their longevity in the BM may contribute to the development of more effective immunosuppressive therapy.

As a diagnostic method, it is necessary for AMR control to establish a humoral immune monitoring method to evaluate AMR pathology in more detail and predict the development of AMR before antibodies, which induce AMR development, are detected in the serum (71, 72). In addition, it has been reported that the antigen specificity of the progenitor cells of LLPCs can be determined using an *in vitro* assay system (22–27), but there are no unambiguous criteria for predicting whether antibodies against donor-specific HLA antigens cause humoral immunity-mediated injury in transplanted grafts (73–75). It is therefore necessary to develop a method that evaluates the reactivity of the antibody to transplanted grafts.

## Development in AMR Control

### Agents Targeting Complement System

The involvement of the complement system in the development of AMR has been reported. In the field of transplantation, the effects of C1-q–positive DSAs on the development of AMR and the incidence of glomerulopathy, as well as the prognosis of transplantation outcomes, have been reported administration of the humanized anti-C5 monoclonal antibody eculizumab, a C5 inhibitor, inhibits the cleavage of C5 to C5a and C5b and the formation of the membrane attack complex C5b-9, and this drug is effective against acute AMR, as indicated by its effective improvement of histopathology in lung transplantation (76–79). In immunologically high-risk cases, the incidence of biopsy-proven AMR following heart or kidney transplantation was significantly lower than that achieved using conventional antibody reduction therapy (80–83).

In addition, C1 esterase inhibitor (C1 INHs) effectively prevents ischemia reperfusion injury (IRI)/delayed graft function (DGF), which has been reported to be involved in the development of AMR through B cell activation, DSA development, and C1q-positive DSA production (84–86). Thus, C1 INHs can be expected to be effective in the maintenance of transplanted graft function by suppressing the aforementioned processes (87, 88). In contrast, no significant inhibitory effects on DSA production and the development of chronic AMR were observed in long-term DSA-positive patients, and no therapeutic effect on AMR was observed in patients with complement-negative DSA (86). Thus, this complement inhibitor should be considered in combination with antibody reduction therapy for AMR control, and it has been reported that transplanted renal graft function and pathological findings are improved by treatment with the standard AMR treatments such as plasmapheresis, IVig, and human plasma-derived C1 INHs (88, 89).

The classic complement pathway inhibitor anti-C1s inhibits C4d deposition, but it does not affect DSA levels and graft function or significantly improve the pathological findings associated with AMR control and clinical prognosis (90, 91). Therefore, it is necessary to elucidate the mechanism by which these complement inhibitors participate in the development of AMR in more detail and improve treatment outcomes in consideration of the administration time and method.

### Agents Targeting Costimulatory Signaling

As a clinical potential of agents targeting costimulatory signaling, the fusion protein belatacept comprises the Fc fragment of human IgG1 linked to the extracellular domain of CTLA-4 and inactivates T cells selectively (92).

In immunosuppressed patients who received of administration of belatacept, their survival and function of their grafts are significantly higher after 7 years of kidney transplantation (93). Compared with cyclosporine-based immunosuppression, this therapy is efficacious for maintaining the function of the transplanted grafts, reduces cardiovascular complications, improves the metabolic profile, and mitigates posttransplant lymphoproliferative disease associated with Epstein-Barr virus infection 1–2 years after transplantation (94). In mice model, an example of an immunosuppressive therapy that may effectively treat DSA-specific MBCs is provided using findings collapsing the GC in a mouse model 7 and 14 days after allo-sensitization directly involves B cells, independent of intervention by graft-specific CD4^+^ Tfhs. Therefore, administration of CTLA- 4 Ig may exert a therapeutic effect on antigen-challenged B cells (95). In contrast, belatacept inhibits the expansion of the Tregs population, which is mediated through the methylation of CpG islands within the Treg-specific demethylation region of the gene encoding FoxP3 (96, 97).

The CTLA-4 Ig fusion protein abatacept, which inhibits CD28-mediated costimulatory signals by binding to CD80/CD86 on the surface of APCs, effectively suppresses T cell activation and cytokine production, which are associated with the development of rheumatoid arthritis (98–100). In the field of transplantation, the function of the transplanted graft persists, and no obvious immunological consequences are observed through the conversion from treatment of a CNI early after transplantation (100).

**Figure 2:**
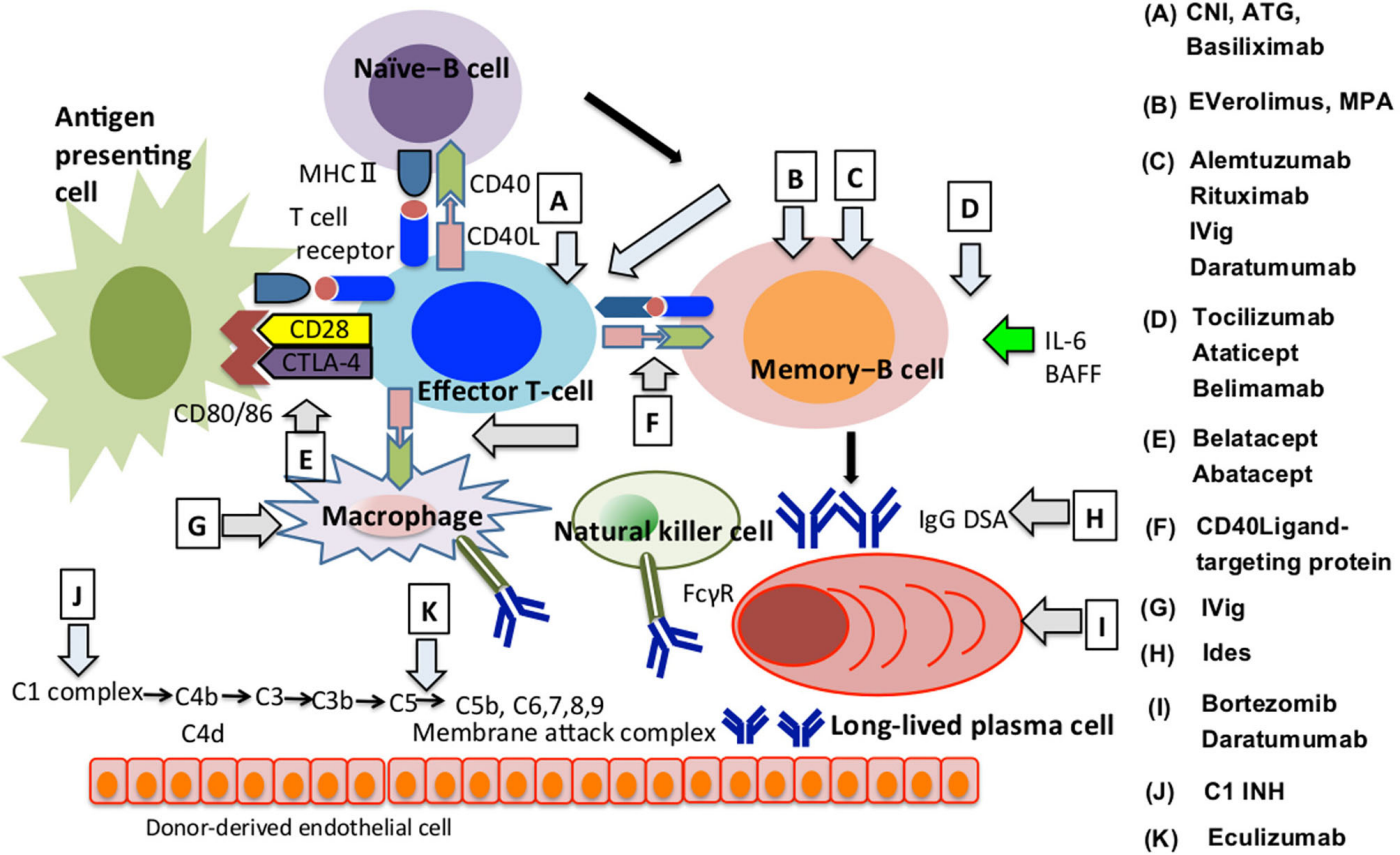
How immunosuppressive agents influence the AMR development. We summarised how immunosuppressants are involved in regulating AMR onset at each B cell differentiation stage, direct or indirect involvement of T cells, macrophages or natural killer cells and the complement system. The alphabet represents immunosuppressants that are effective in the differentiation process indicated by arrows. AMR, Antibody-mediated allograft rejection; ATG, Antithymocyte globulin; BAFF, B Cell Activating Factor; CD40L, CD40 ligand; CTLA-4, Cytotoxic T-lymphocyte associated antigen 4; DSA, Donor-specific HLA antibody; FcγR, Fc gamma receptor; C1 INH, Human plasma-derived C1 esterase inhibitor; CNI, Calcineurin inhibitor; Ides, IgG-degrading enzyme of *streptococcus pyogenes*; IVig, Intravenous Immunoglobulin; MHC, Major histocompatibility complex; MPA, mycophenolic acid.

Action of conventional immunosuppressive therapies and these novel therapies targeting the complement system and co-stimulatory signaling in immune system and their clinical potential for AMR control, their clinical potential for AMR control, are presented in **Table 1** and the pathways of differentiation of naïve-B cells into LLPCs and the immunosuppressive agents that influence these processes are shown in **Figure 2**. In addition, the involvement mechanism and clinical potential of cytokines involved in the differentiation of naïve-B cells into LLPCs in the context of AMR are summarized in **Table 2A**, and the clinical applicability of these molecules and possible use in AMR control are summarized in **Table 2B**.

**Table 1:** Effects of immunosuppressive therapy on the immune system and its clinical role in AMR control.

	Involvement mechanism in Immune system	Clinical role for AMR control	Reference
	Effects on The Cell Cycle		
Everolimus	Inhibition of cell division, cell proliferation, and angiogenesis through inhibition of the phosphorylation of mammalian targets of rapamycin and formation of a complex with the FK506-binding protein (FKBp)-12	Increased risk for the development of DSA and AMR by evelolimus-based immunosuppression Increased risk for the development of *de novo* DSA by early conversion of CNIs to everolimus No effect on the risk of *de novo* DSA development by late conversion of CNIs to evelolimus Induction of Tregs	(101–105)
Mycophenolic acid	Inhibition of DNA synthesis in lymphocytes through inhibition of the activity of IMPDH 2 and reduction of the sizes of intracellular pools of guanosine nucleotide	Reduction of anti-HLA class I and II antibody production Improvement of patient and graft survival and reduction of rejection episode while using with CsA and steroids	(106–108)
	**Effects on Molecules Expressed By B Cells**		
Alemtuzumab: A humanized anti-CD52 antibody	Induction of B cell apoptosis through binding to CD52, which is frequently expressed by B cells	Reduction of incidence of *de novo* DSAs and AMR development by Alemtuzumab induction therapy Reduction of the risk of AMR by using Alemtuzumab induction therapy combined with belatacept and rapamycin	(109–111)
Rituximab: Anti-CD20 monoclonal antibody	Induction of CD20 (+) B cell apoptosis through its binding to CD20, which is found on mature B cell	Improvement of survival in cardiac allograft AMR Reduction of DSA levels and microcirculation inflammation after late AMR by using with sterorid/Ivig	(112–117)
	**Effects on Antibody-producing Cells and Antibody**		
Bortezomib	Induction of apoptosis of antibody-producing cell through inhibiting the proteasome	Maintenance of renal graft survival after late occurrence of AMR with high probability using combined with rituximab and methylprednisolone, and plasmapheresis Reduction of DSA levels and prevention of AMR in sensitized patients with crossmatch-positive and elevated DSA in cardiac transplant	(118–120)
IdeS	Removal of anti-HLA antibodies through the cleavage of IgG at a specific amino acid sequence within the hinge region and reduction of antibody-producing cells	Reduction of anti-HLA antibodies level	(121, 122)
	**Effects on Antibody-Receptor**		
IVig	Induction of mature B cell apoptosis Suppression of proinflammatory cytokine production such as that of TNF-a through the inactivation of macrophages mediated by FCγR blocking	Reduction of DSA level and C4d deposition intensity after acute AMR using plasmapheresis and repeated infusions of IVig	(71, 123, 124)
	**Effects on T Cells**		
CNIs	Inactivation of the calcineurin-dependent NFAT pathway and T cells through the formation of a complex with cyclopherin or FK506	Prevention of *de novo* DSA formation	(102, 125–128)
rATG	Induction of T cells depletion	Depletion of DSAs No effect on the vascular AMR outcome and transplant prognosis improvement	(129–131)
Basiliximab: mouse-human chimeric monoclonal antibody	Induction of T cells depletion through reaction with the α-chain (CD25) of the IL-2 receptor expressed by T cells	Prevention of the rejection development, especially in kidney transplantation	(132)
	**Effects on The Costimulatory Signaling**		
Belatacept; CTLA- 4 Ig	Reduction of antigen-challenged B cell Inactivation of T cell. Inhibition of Treg expansion	Depletion of plasma cells producing DSA and reduction of DSA levels in active AMR	(92–107)
Abatacept: CTLA- 4 Ig	Inactivation of T cell through inhibition of CD28-mediated costimulatory signals by binding to CD80/CD86 on the surface of APCs	Extension of graft survival with combined bortezomib use in a sensitized animal kidney transplant model	(98–100)
	**Effects on The Complement System**		
Eculizumab: humanized anti-C5 monoclonal antibody	Blocking membrane attack complex formation and its function	Improvement of histopathology and transplanted graft function and prevention of early active or chronic AMR development in positive crossmatch HLA incompatible patients	(76–79, 133, 134)
C1 INH: human plasma-derived C1 esterase inhibitor	Inactivation of C1r and C1s proteases in the C1 complex of classical pathway of complement.	Improvement of histopathology and graft survival with the combined use of plasmapheresis and IVig Prevention of AMR development through IRI/DGF prevention	(84–89)
Anti‐C1s: Classic complement pathway inhibitor	Inhibition of complement pathway	No significant effect on graft outcome and histological findings Reduction of C4d deposition	(90, 91)
	**Anti-inflammatory Effects**		
Glucocorticoids	Downregulation of the expression of AP-1 and NF-κB	Maintenance of renal allograft survival after combination of bortemizob, corticosteroids, rituximab, and plasma pheresis for late onset AMR	(135, 136)

AP-1, Activator protein1; AMR, Antibody-mediated allograft rejection; APC, Antibody producing cell; CNI, Calcineurin inhibitors; CsA, Cyclosporine; CTLA-4, Cytotoxic T-lymphocyte(associated)antigen 4; DGF, Delayed graft function; DSA, Donor-specific HLA antibody; FcγR, Fc gamma receptor; HLA, Human Leukocyte Antigen; Ides, IgG-degrading enzyme of streptococcus pyogenes; IMPDH2, Inosine-5’-monophosphate dehydrogenase 2; IRI, ischemia reperfusion injury; IVig, Intravenous immunoglobulin; MMF, Mycophenolate mofetil; NFAT, Nuclear factor of activated T-cells; NF-κB; Nuclear factor κB; rATG, Recombinant Anti-thymocyte globulin; SRL, Sirolimus; TNF, Tumor Necrosis Factor; Treg, Regulatory T cell.

**Table 2A:** Involvement of cytokines in the immune system and clinical applicability for AMR.

Cytokine	Involvement of cytokines in the immune system	Clinical applicability for AMR control	Reference
IL-2	Plasma cell generation	Extension of heart allograft survival by IL-2 gene expression inhibition in a mouse model	(137–139)
IL-6	Support of B cell growth and survival including isotype switching, spontaneous germinal center formation, and IgG production Induction of IL-10-producing B cells Induction of Th 17 cell differentiation	Tocilizumab (anti-IL -6 receptor monoclonal antibody) showed significant improvement in graft survival, function, and DSA reduction 6 months after the treatment in chronic active AMR Clazakizumab (anti‐IL‐6) showed significant reduction of DSA levels and the suppression of AMR activity, progression	(140–149)
IL-7	Support of B cell development, immunoglobulin gene rearrangement Induction of Th17 cells Extension of the function of FoxP3 (+) natural regulatory T cells	Anti-IL-7 monoclonal antibody targeting IL-7 receptor α showed extension of allograft survival and induction of allograft tolerance in heart transplants and promotion of long-term allograft survival by IL-7 inhibition in combination with T cell depletion synergized with either CTLA-4 Ig administration or tacrolimus in pancreatic islet allografts	(150–153)
IL-10	Down-regulation of antigen-specific T cell response	Circulating IL-10 (+) Breg levels indicate the AMR resistance after kidney transplantation	(154–161)
Il-15	Support of B cell proliferation and antibody production Induction of regulatory CD8 (+) CD122 (+) T cell and NK cell-derived IFN-gamma Inhibition of pathogenic Th17-cell differentiation,	Antagonistic mutant IL-15/Fc fusion protein (mIL-15/Fc) is effective in the prevention of allograft rejection induce antigen-specific tolerance in minor histocompatibility complex-mismatched recipients and extend cardiac allograft survival in fully MHC-mismatched recipients IL-15 is a biomarker of acute and chronic allograft rejection Anti-IL-15 therapy is effective in the prevention of acute and chronic allograft rejection using classic immunosuppression	(162–164)
IL-21	Support of plasma cell differentiation, Support of IL-10-producing regulatory B cells differentiation	The administration of IL-21 receptor fusion protein (R-Fc) prevents chronic cardiac allograft vasculopathy in a heart allograft transplant mouse model Frequency of donor-specific IL-21 producing cells is effective as a biomarker for the prediction of rejection	(165–167)
IL-35	Induction of IL-10 producing B cell Expansion of regulatory B cell and regulatory T cell Antagonizing Th1/Th17 responses	IL-35 gene therapy prolonged graft survival in a mouse heterotopic abdominal heart transplantation model combined with a methyltransferase inhibitor treatment	(154–161)
TNF-α	Augmentation of B-cell proliferation, polyclonal B-cell, B cell malignancies Development of germinal center B cell Generation of extra follicular T-bet (+) B cell	Serum level of TNF-α is associated with histologically findings and is effective as a biomarker for AMR development	(168–170)
TGF-beta	Induction of immune tolerance Inhibition of antibody production Enhancement of FoxP3 and CTLA-4 expression in Tregs	Anti-TGF-beta antibody treatment significantly reduces chronic rejection and prevent dysfunction of renal allografts in rats	(48, 154–161)
BAFF	Promotion of B cell growth and survival, and antibody production Maintenance of survival of high-affinity B cell clones	Belimumab, a human monoclonal antibody that inhibits BAFF, removes complement-binding anti-HLA class I and class II antibody in pre-HLA sensitized patients Elevation of perioperative BAFF level predicts the risk of acute AMR development The BAFF mRNA expression level significantly unregulated in chronic AMR compared with graft function stable and healthy donors in renal transplants	(171–174)

AMR, Antibody-mediated allograft rejection; BAFF, B Cell Activating Factor; CTLA-4, cytotoxic T-lymphocyte(associated)antigen 4; DSA, Donor-specific HLA antibody; HLA, human leukocyte antigen; IFN, Interferon; IL, Interleukin; MHC, major histocompatibility complex; NK cell, Natural killer cell; TGF-beta, Transforming Growth Factor-beta; Th, T helper; TNF, Tumor Necrosis Factor.

**Table 2B:** Molecules expressed by B cells and clinical applicability for AMR control.

Molecules	Involvement mechanism in Immune system	Clinical applicability for AMR control	Reference
CD38	Support of B cell activation and proliferation as co-receptors for cytokine receptors and inhibit apoptosis of GC B cell through phosphorylation of CD19 Reduction of plasma cells in the BM	Daratumumab (humanized, CD38‐targeting antibody) reduce DSA level rapidly and extent graft survival	(175–179)
CD40	Support of proliferation and survival B cell through CD40/CD40 ligand interaction	Inhibiting signaling through the CD40/CD40 L pathway inhibits B cell activation, suppresses plasma cells differentiation, and suppresses TD antigen-specific IgG production Blocking the CD28/B7 and CD40/CD40L interaction at the same time delay or prevent allograft rejection	(180, 181)
TACI	Inhibition of B cell expansion. Regulation of serum BAFF level Promotion of GC B cells apoptosis Promotion of plasma cells survival and differentiation, and antibody production	Atacicept is effective in the reduction of DSA levels and extension of graft survival TACI mRNA expression level significantly unregulated in chronic AMR compared with graft function stable and healthy donors in renal transplants	(172, 182–185)
BCMA	Induction of the antigen presentation response Support the survival of late memory-B cell and all plasma cells by binding APRIL	Elevation of BCMA level is an effective biomarker for the development of *de novo* alloantibody responses in a mouse skin allograft transplant mouse model BCMA mRNA expression level significantly unregulated in chronic AMR compared with graft function stable and healthy donors in renal transplants	(66, 172, 186–189)

APRIL, A proliferation inducing ligand; AMR, Antibody-mediated allograft rejection; BAFF, B Cell Activating Factor; BCMA, B-cell maturation antigen; BM, Bone marrow; CD40L, CD40 ligand; DSA, Donor-specific HLA antibody; GC, Germinal center; TACI, Transmembrane activator and calcium-modulating cyclophilin ligand interactor; TD, Thymus-dependent.

### Agents Targeting Molecules Expressed in B Cell

In addition to these listed molecules, CD19-mediated signaling reduces the threshold for BCR-mediated signal and promotion of B cell development (190). Under pathological conditions, inebilizumab (humanised anti-CD19 monoclonal antibody) significantly reduced autoantibodies and relapse rates in patients with neuromyelitis optica (191).

Although antibodies targeting CD20 are used for the treatment of AMR, CD19 is expressed at all B cell differentiation stages, is more widely expressed than CD20 and is expressed in PCs. Therefore, CD19-targeting therapy may be more useful than CD20-targeting therapy in AMR treatment (19, 20) and the safety and tolerability of inebilizumab alone or in combination with VIB4920 (Fc-deficient CD40 Ligand antagonist) are currently being investigated in highly sensitised patients on the waiting list for kidney transplantation at clinicalTrials.gov (study identifier NCT04174677) (192).

CD138, a member of the integral membrane family of heparan sulfate proteoglycans, is highly expressed on PCs (193). CD138 increases heparan sulfate levels in ASCs, and APRIL and IL-6 support the growth and survival of ASCs by binding to heparan sulfate (194).

In other words, CD138 plays an important role in the maintenance of long-term humoral immunity (195).

In the field of transplantation, the relative abundance of CD138-positive cells is closely related to AMR development, the degree of humoral immunity-associated injury progression, and DSA production (196), and a higher frequency of CD138-positive cells and the co-existence of CD20-positive cells are associated with poor allograft function and poor response to treatment after acute rejection including TCMR and AMR (197). In addition, pathological findings indicate that the CD138-positive PCs infiltrates are associated with AMR development (196). Therefore, CD138-targeting therapy may be applicable for AMR control and the maintenance of graft function after further elucidation of the involvement mechanism of CD138-mediated signaling in the activation of humoral immunity to transplanted grafts (43). However, as described in B−cell based therap*y*, B cells contain a subset that has an immune regulatory function. Therefore, any clinical application for AMR control needs to be fully examined for the susceptibility of these agents for each of the B cell subsets, and the effect it will have upon their function.

### B-Cell Based Therapy

In this section, we will discuss B cell subsets with immunoregulatory function because the expansion of regulatory cells that induces immune tolerance that may lead to the reduction of immunosuppressants.

Bregs mediate immune tolerance through mechanisms that involve the production of cytokines such as IL-10, IL-35, and TGF-beta as well as through cell–cell contact (154–161). Although IL-10 has been the focus of attention, IL-10 and IL-35 produced from Breg play important roles as negative regulators of immunity, which are involved in the antigen-presenting function and cytokine secretion of macrophages, dendritic cells, and B cells and activation of inflammatory T cells (156). B-1 cells and marginal zone (MZ) B cells exhibit innate-like immune functions (198). MZ-autoreactive B cells are required to maintain tolerance to self-antigens and the rapid acquisition of immunoregulatory function through the maintenance of the levels of natural IgM antibody and IL-10 (199, 200). Furthermore, ILBs serve as important sources of Bregs that play crucial roles in autoimmunity, inflammation, and infection in the models of autoimmune disease (201, 202).

As other B cell subsets that have been reported to be involved in the induction of immune tolerance, naïve-B cells function as APCs and induce the conversion of CD4^+^ CD25^−^ T cells into CD25^+^ Foxp3^−^ Tregs, which express lymphocyte activation gene 3 (LAG3), ICOS, glucocorticoid-induced TNF receptor family-regulated gene (GITR), OX40, PD1, and CTLA-4, to sustain immune tolerance through the production of anti-inflammatory cytokines such as IL-10. These findings indicate that naïve-B cells cooperate with Tregs to induce T cell tolerance, maintain the homeostasis of Tregs, and suppress inflammation by functioning as APCs (203, 204). In addition, IgM^+^CD138^hi^TACI^+^CXCR4^+^CD1d^int^Tim1^int^ PCs expressing the transcription factor Blimp1 produce IL-10, IL-35 during infections with *Salmonella* species. Furthermore, CD138^+^ PCs provide the major source of IL-35 and IL-10 in patients with experimental autoimmune encephalomyelitis (EAE) (156).

In the field of transplantation, IL-10 produced by Bregs is associated with drug resistance in AMR, and higher frequencies of transitional B cells and naïve-B cells and high production of IL-10 are related to the induction of immune tolerance following kidney transplantation. In contrast, the results are opposite in cases of chronic AMR (205), and CD138^+^ PCs may be involved the induction of immune tolerance and the expansion of these B cells may mitigate the adverse effects of immunosuppressants and thereby improve the prognosis of patients who undergo transplantation.

## Development of Diagnostic Methods

### Monitoring Humoral Immunity Using Memory-B Cell

The MBC pool has recently attracted more attention than humoral antibodies as a potential diagnostic tool to monitor the humoral immune response to donor-specific HLA antigens, which is evaluated according to the frequency of DSA-specific MBCs circulating in peripheral blood. For example, to determine the antigen specificities of MBCs circulating in the periphery, B cells or peripheral blood mononuclear cells (PBMCs) are induced to differentiate into ASCs with mitogens and cytokines suitable for the proliferation of MBCs and survival in a polyclonal activation-dependent manner. These findings were acquired through analysis of culture supernatants using a solid-phase assay platform with HLA-coated multiplex beads (23–29).

In the field of transplantation, current studies focus on the diagnostic value of IgG antibodies against HLA expressed in donor-derived vascular endothelial cells. On the other hands, there is currently no consensus on the clinical role of IgM antibodies (206).

We examined the clinical potential of DSA-specific IgM-MBCs as early diagnosis and humoral immune monitoring in the context of AMR; DSA-specific IgM-MBCs may achieve higher sensitivity when employed for conventional immunosuppressive therapy compared with IgG-MBCs (207). Therefore, the detection of DSA-specific IgM-MBCs contributes to the inhibition of AMR development by enabling early intervention using less invasive immunosuppression after elucidation of the optimal conditions for inducing DSA-specific IgM-MBC differentiation into IgG-PCs, the process that leads to AMR. In addition, the availability of an *in vitro* assay system capable of inducing the differentiation of MBCs into ASCs may lead to the introduction of more effective immunosuppressive therapy that recognizes differences in pathology or in patients’ drug sensitivities (208).

However, it is unclear whether *in vitro* drug susceptibility data can be applied to patients because conventional two-dimensional culture may not reproduce the three-dimensional structure or function such as lymphoid tissue or organs in the living body. Organ-on-a-chip technology is attracting attention because it reproduces organs in functional units while maintaining the *in vivo* three-dimensional organ structure and physiological function. Thus, pharmacokinetic analysis using this model may be useful for resolving the aforementioned problem (209–212).

### Microarray Technique

Attempts are being made to apply microarray technology or NGS to the field of transplantation. Microarrays simultaneously analyze the expression of tens of thousands of genes and obtain information about the transcriptional profiles. Furthermore, pathological changes in gene expression can be obtained, which will provide information to increase the accuracies of classification, diagnosis, and prognosis of diseases (28, 29). Errors frequently occur in analyses of single genes when small data sets are employed. Recently, Chen et al. focused on transcripts that are known to be associated with disease. Microarray evaluation of pathogenesis-based transcript sets, corresponding to events that mainly occur during allograft rejection, is useful for the diagnosis of AMR as well as identification of pathology and prediction of clinical course. For example, biopsy-based microarrays identified 45 genes upregulated in pediatric kidney transplants as well as in pediatric and adult heart transplants undergoing acute rejection. Among them, serum PECAM1 shows the greatest promise as a biomarker (89% sensitivity and 75% specificity) for analyzing renal transplant rejection, suggesting that microarray analysis will be useful for discovering new serum protein biomarkers by mining publicly available data sets (30). Thus, it is possible to identify the gene group associated with graft rejection and pathological condition by enabling comprehensive gene analysis; early diagnosis of the development of rejection is possible and genes related to immune status or drug resistance has also been identified (213).

### Next-Generation Sequencing

NGS determines several million base pairs per run and provides the ability to distinguish different isoforms and allelic expression, which is an advantage over microarray analysis and detects somatic mutations with high accuracy and high specificity, which enables identification of candidate genes that cause disease. As an example of the application of NGS to transplantation, comparison of the complementary determining region 3 (CDR3) of the TCR beta chains expressed in AMR (+) and AMR (−), may lead to the prediction of the development of rejection before patients undergo transplantation (31). In addition, this technique can be applied to increase our understanding of the diversity of the variable regions of heavy and light chains of BCRs. Thus, detailed characterization of this repertoire, including reactivity with antigen, becomes possible and may be applied to predict the production of donor-specific antigen-reactive antibodies (32–34) and both specific sequences and the full length of genes including introns and untranslated regions are analyzed by NGS, and thus, HLA alleles can be analyzed in transplantation, ensuring better histocompatibility between donors and recipients (214, 215).

In addition, gene polymorphisms affect the distribution and drug metabolism. For example, Single Nucleotide Polymorphism (SNP) of CYP3A4/3A5, ABCB1 in CNI, CYP3A5 in sirolimus, TPMT in azathioprine, UGT1A9, ABCC2 in Mycophenolic acid (MPA), and MDR1 in tacrolimus affects their pharmacokinetics. Thus, NGS analysis of these SNP will likely provide useful information for developing more effective immunosuppressive therapy, which considers differences in pathology and drug susceptibility (35, 36).

In the other words, these techniques have potential application in developing strategies for controlling AMR and improving the prognosis of transplanted grafts.

## Novel Appliable Components in AMR

As mentioned above, we are closely evaluating the possibility of using an IgM antibody in AMR control (207). Therefore, we evaluate its possible use not only as an early diagnosis tool but also as a treatment method. Here, we discuss the involvement, as well as their clinical applicability of humoral immunity-associated components involved in IgM antibody in AMR control whilst referring to reports on the involvement mechanisms in pathological conditions in fields outside of transplants.

### Role of IgM Receptors

IgM is the first immunoglobulin produced in response to antigen challenge, and B cell activation is enhanced by stimulatory IgM Fc receptor (FcμR)-mediated signals during the early stage of the immune response. The levels of IgG exceed those of IgM during the late stage of the adaptive immune response, during which the activation of B cells is inhibited by FcγRIIB-mediated inhibitory signals, indicating that stimulatory or inhibitory signals transduced by IgM or IgG may regulate B cell activation and antibody production (216).

FcμR is mainly expressed by B, T, and NK cells in humans and by B cells of mice (216–219). The expression levels of mouse FcμR differ depending on the B cell subset, and FcµR expressed in the trans-Golgi network restricts the transport of IgM-BCR to the B cell surface. FcμR expression is downregulated during GC reactions and at a higher level in plasma blast cells compared with those expressed by PCs and expressed in class-switched B cells (187). In contrast, FcμR-deficient MZBs are significantly mitigated in a mouse knockout model, in which the number of B cell subsets is altered or tonic signaling through the reduction of BCR expression (216, 219, 220). The FcμR-mediated signal contributes further to the maturation of B cell subsets and enhances B cell survival in response to treatment with anti-IgM antibodies (216, 221). These findings indicate that signals transmitted by the FcμR and BCR may cooperate to activate B cells and maintain their survival. In addition, this signal regulates the expression of IgM-BCR in immature B cells, which regulates the signal emitted when antigen activates the BCR through their cross-linking by antigen binding (220). In FcμR KO mice, the levels of natural IgM antibodies in the peripheral blood circulation are increased, and the formation of the GC, MBCs, differentiation of PCs, and production of antigen-specific IgG1 antibodies are reduced. These findings support the conclusion that the FcμR is required for the maintenance of the adaptive immune response and the control of the homeostasis of B-1/B-2 cells (216, 219).

Under pathological conditions, FcμR-mediated signaling controls the production of harmful autoreactive IgG antibodies and is involved in the development of autoimmune and inflammatory diseases, chronic lymphocytic leukemia, and others (37). In a model of severe human multiple sclerosis, the development of EAE is suppressed through the regulation of the functions of dendritic cell and Tregs (222). FcμR-mediated signal transduction increases self-Ag-triggered BCR signaling in immature B cells and contributes to the deletion, anergy, or both of autoreactive immature B cells in the BM, which induces immune tolerance (38).

Soluble IgM is a ligand for CD22 and forms a complex with an antigen. This complex suppresses the CD22-mediated BCR signaling *via* its binding to CD22 expressed on the B cell surface. CD22, which is expressed on the surface of mature B cells, is an inhibitory receptor. Specifically, phosphorylated CD22 signals through the BCR to downregulate B cell activation that prevents the overactivation of the immune system and development of autoimmune disease (223, 224).

In CD22-deficient mice, BCR ligation promotes the mobilization of intracellular calcium and inhibits BCR signaling. Furthermore, CD22 signaling contributes to the differentiation of B cells and is required for the expansion of B1-b cells after BCR ligation (225, 226). The response to the TD antigen is normal during adaptive immunity; however, Thfs do not support B cells in CD22-deficient mice, because the CD22-CD22-ligand (CD22L) interaction is required for the activation of T cells (224, 226). In CD22-deficient mice, naïve-B cells differentiate into GCB cells but cannot differentiate into MBCs or PCs (221, 222). CD22-mediated inhibition of the BCR signal is associated with B cell tolerance, and CD22-CD22L interactions are required to maintain self-tolerance (227, 228).

Under pathological conditions, CD22 is involved in the control of autoimmune diseases and genetic variants of CD22 are related to the susceptibility of individuals to autoimmune diseases through a defect in B cell tolerance (39, 40). In patients with autoimmune diseases such as rheumatism, T1-diabetes, and SLE, inactivating mutations are frequently found in the CD22-ligand (229–231). Although CD22 is not a major cause of susceptibility to SLE in humans, CD22 deficiency may exert additive or synergistic effects on susceptibility to disease. Moreover, CD22 regulates the B cell response in autoimmune disease through regulation of BCRs and Tool-like receptors (TLRs) (224, 232).

### Role of Scavenger Protein

Accumulation of foreign pathogens, apoptotic or necrotic dead cells, and their debris causes chronic inflammation and induces an autoimmune response, which must be eliminated to prevent their onset.

Apoptosis inhibitor of macrophage (AIM, also called CD5L) is a circulating protein that is a member of the scavenger receptor cysteine-rich superfamily. Normally, high levels of AIM bind to IgM pentamers and circulate in the peripheral blood in the inactivated state (233).

In B cells, AIM cooperates with TGF–beta1 to suppress B cell proliferation strongly and persistently and inhibit antibody production. TGF-beta1-mediated increased expression of AIM receptors on the surface of B cells is required for AIM to exert an effect on these cells (234). In addition, it has been reported that IL-10 protects transplanted grafts from recipient immunity and exert anti-inflammatory effects by inhibiting NLRP3 inflammasome activation or MPs with proinflammatory phenotypes by increasing AIM expression. Therefore, regulation of AIM expression may be related to the induction of immune tolerance (35).

Under pathological conditions, IgM dissociates from AIM during the recovery from renal injury through the enhanced clearance of a luminal obstruction during acute renal injury (234–236). Specifically, free AIM bound to debris interacts with kidney injury molecule 1 (KIM-1) that is expressed by injured tubular epithelial cells, resulting in enhanced phagocytic clearance of AIM-bound debris by epithelial cells (234, 237–239). Thus, free AIM is involved in the repair of acute kidney injury, because delayed or deficient removal of dead cells, or both, may cause secondary inflammation and fibrosis in tissues and may impair the repair of tissue damage and the regeneration of such tissues (237). Alternatively, macrophages produce AIM (236), which is involved in the pathophysiology of inflammatory colitis through the maintenance of the survival of macrophages, and the elimination of dead cells and toxic substances in hepatitis by supporting the phagocytic activity of macrophages (239–241). In the field of transplantation, it has been reported that the blood concentration of free AIM increases during acute cellular rejection in cardiac allograft rejection (242).

### Role of IgM Antibody

Natural IgMs exert anti-inflammatory effects through clearing pathogens, scavenging toxins, inhibiting the production of inflammatory mediators, neutralizing cytokines, and scavenging complement to directly protect antigens from humoral immune attack (207, 243–248). These antibodies suppress inflammation and injury of target tissues caused by IgG autoantibodies through anti-idiotype activity, competitive inhibition of binding of IgG autoantibody to antigens, and suppression of IgG autoantibody production *via* signals from the FcµR expressed on the surface of B cells (249). Furthermore, these antibodies significantly inhibit T cell proliferation in lectin-stimulated PBMCs *in vitro* through the suppression of IL-2 production that enhances the functions of immunocompetent cells, bacterial aggregation, and opsonic activity (37). A polyclonal antibody preparation designated trimodulin, which contains IgM (~23%), IgA (~21%), and IgG (~56%), decreases the levels of TLR2, 4 as well as those of coagulation receptors (CD11b and CD64) in monocytes and inhibits lymphocyte proliferation and regulate of the production of pro- and anti-inflammatory cytokines, including TNF-α and IL-10. Therefore, these antibodies achieve a protective effect to alleviate the inflammatory reaction to target organs through these extents of involvement in immune system (243, 246, 247, 250–252).

In a model of renal ischemia-reperfusion-induced injury, the tissue-protective effects of IgM antibodies that recognize and inhibit the activities of danger-associated molecular patterns reflect human pathology. These mechanisms support the regeneration of damaged hepatocytes in a model of liver ischemia (42). In patients with sepsis, IgM-enriched IVig ameliorates pathology and reduces mortality through the improvement of the peripheral circulation, such that the numbers of circulating B cells and levels of IgM are significantly reduced in nonsurvivors compared with survivors, indicating that IgM is required to achieve successful therapy (253–256).

In an *in vitro* model of xenotransplantation, compared with IVig, IgM-enriched IVig more strongly inhibits the classical complement pathway and complement-dependent cytotoxicity caused by the deposition of C4 and C3 on the cell surface of pig cells treated with human serum and suppresses the development of hyperacute rejection of a xenotransplanted graft (257).

In allograft transplantation, IgM inhibits complement activity 10-times more than IgG (258). In the case of *de novo* DSA production after lung transplant recipients; treatment with IgM-enriched IVig is associated with the DSA-clearance effect, which improves the prognosis of transplantation. In early DSA-positive cases, treatment with IgM-enriched IVig and rituximab was superior to that with plasma exchange and rituximab, and the patients were comparable to the DSA-negative group in 1-year survival after transplantation; treatment with IgM-enriched IVig was superior to treatment with plasma exchange and rituximab in DSA clearance (259). In addition, a tissue biopsy confirms no AMR after treatment only by IgM-enriched IVig injection, when AMR develops after heart transplantation (258).

### Clinical Potential of Humoral Immunity–Associated Components in AMR

Further elucidation of the involvement of AIM in the humoral immune response to transplanted grafts will be applicable for AMR control. Newly discovered cytokines, antibodies, and receptors involved in antibody production may be expected to be translated to the clinical application in early diagnosis, management, and prognosis prediction. These efforts require the identification of the roles of these components in the underlying mechanisms of AMR.

In the field of transplantation, FcμR-mediated signaling maintains the homeostasis of B1/B2 cells (216, 219) and potentially alleviates the pathology of AMR (38), and elucidation of the involvement of this signaling in AMR development will likely contribute to the development of therapies designed to protect target grafts from humoral immune responses mounted by recipients.

CD22-mediated signaling may be expected to be useful as B cell depletion therapy in AMR control, because IVig is administered as a treatment for AMR, and one of the mechanisms is the induction of mature B cell apoptosis *via* binding between sialylated IVig and CD22 (71). It is therefore important to evaluate the involvement of CD22-targeting agents in the activation of humoral immunity against transplanted grafts in the same manner as they are applied to the treatment of autoimmune diseases to effectively manage AMR (231, 232).

As a diagnostic method, clinical application of these receptors to new diagnostic methods will be achieved if the functions and structural properties of these receptors can be shown to be involved in humoral immunity to transplanted grafts.

As scavenger protein, it has also been reported that an increase in AIM blood concentrations is associated with the development of acute rejection following heart transplantation, indicating that the blood concentration of free AIM may increase in the early stage of humoral immunity activation in transplanted grafts, and it is expected to be useful as an early diagnostic method for AMR (242). Therefore, the utility of AIM as a diagnostic modality can be ascertained after elucidating its AIM in the development of AMR. In addition, AIM controls inflammation and repairs damaged tissues *via* phagocytosis of apoptotic cells.

Although IVig has been commonly used for AMR control, the significance of IgM-enriched IVig has been reported in the field of severe infection and organ ischemia (42, 253–258). IgM-enriched IVig may exert more strongly inhibits complement activation as compared with IVig (258, 259). Therefore, identifying the detailed mechanism of IgM involvement in humoral immune activation to transplanted grafts and the significance of IgM-enriched IVig compared with that of IVig will likely lead to further clinical application of IgM-enriched IVig to manage AMR.

Significantly, in addition to the potential induction of immune tolerance of humoral immunity-associated components to transplanted grafts (35, 243, 247, 248, 250–252), AIM and IgM-enriched IVig have a tissue repair and regeneration effect through the clearance of injured tissue (42, 224, 233–235). Therefore, humoral immunity associated injury after the onset of AMR, which was considered irreversible, could be recovered by clinical application of these humoral immunity-associated components.

**Figure 3:**
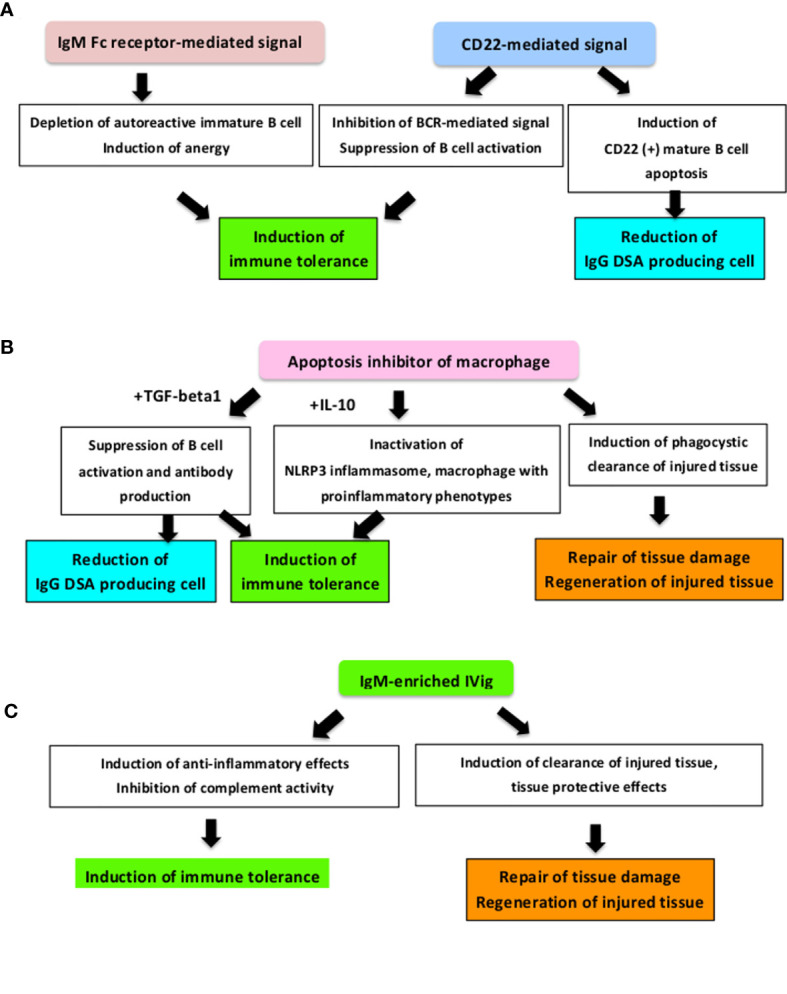
How these novel applicable components improve the prognosis of AMR. We summarised the clinical potential of novel humoral immunity-associated components in AMR control and AMR development mechanisms. **(A)** As an IgM receptor, the FCu receptor-mediated signal controls harmful autoreactive IgG antibodies production and controls autoimmunity and inflammation through regulating immunoregulatory cells (37, 38, 216, 219, 222). In addition, this signal is associated with immune tolerance induction by promoting deletion and energy of immature B cells (38). CD22-mediated signaling also induces immune tolerance *via* CD22-CD22L interaction and CD22-mediated inhibition of the BCR-mediated signal (227, 228) and CD22 (+) mature B cell apoptosis (71). **(B)** As a scavenger protein, it has been reported that free Apoptosis inhibitor of macrophage (AIM) works to suppress inflammation, tissue injury and tissue regeneration through phagocytic activity during acute kidney injury (233–241) and AIM mediates immune tolerance induction by IL-10 and suppresses the immune response by inducing macrophages with immunosuppressive phenotype activation (35) and cooperates with TGF-beta to suppress B cell proliferation and antibody production (233). **(C)** As a polyclonal IgM antibody, IgM-enriched IVig strongly suppresses cell proliferation, has a inhibitory effect in complement activity (257, 258), and alleviates the humoral immunity-associated pathological condition in severe infection, organ ischemia, and transplants (42, 253–256, 258, 259). BCR, B cell receptor; DSA, Donor-specific HLA antibody; IVig, Intravenous immunoglobulin; TGF, Transforming Growth Factor.

We summarized clinical potential of these components in the context of AMR control (**Figure 3**).

## Concluding Remarks

In this study, we discussed the pathways by which naïve-B cells are sensitized to donor-specific HLA antigens and differentiate into LLPCs, which produce DSAs. This study also presented evidence regarding the mechanism by which immunocompetent cells participate in the signal transduction pathways that contribute to AMR and the mechanisms of immunosuppressive therapy designed to suppress the development of AMR (17–19).

Although immunosuppressive therapies that may be useful for suppressing each process during AMR development have been developed, AMR control remains challenging. The main cause of poor AMR control is that positivity for antibodies to donor-specific HLA is used as a reference as one of the diagnostic methods for AMR (17); however, LLPCs, which produce antibodies inducing AMR, may be difficult to remove by applying conventional immunosuppressive therapy and the pathological condition progresses irreversibly when these antibodies are present in serum (20, 71, 72, 260). In addition, the information needed to improve management after transplantation including individual differences in pathological conditions and drug susceptibility has not been discovered.

It has been reported that humoral monitoring methods for better evaluating the humoral immune response to the transplanted graft might contribute to resolving this problem by analyzing the antigenic specificity of MBCs circulating in peripheral blood using *in vitro* assay systems (22–27) and further possibility of gene-based control method in the context of AMR as following; Microarray techniques can be used to elucidate the molecular mechanisms that induce the migration of LLPCs to BM and promote their longevity (259). Thus, these techniques may permit inhibition of the differentiation of LLPCs. Further; NGS analysis of antibody reactivity to transplanted grafts can be applied to predict the antibodies that cause AMR (28–36). This knowledge, combined with the identification of genetic polymorphisms associated with drug sensitivity, will undoubtedly contribute to the development of optimal management strategies.

In addition to the development of these diagnostic methods, recent studies revealed relevant factors, such as anti-inflammatory effects, reduction of harmful IgG autoantibodies production, tissue regeneration, and their clinical applicability in the fields of autoimmune diseases and inflammatory disease and severe infection disease, and the others (37–42). Therefore, in the field of transplantation, by further clarifying the mechanism of their involvement in AMR, methods for improving the control of AMR including early diagnosis, suppression of AMR development, and alleviation of pathology may be developed. 

Although IgG antibody has been the main focus in the field of transplants, we focused on the clinical potential of detecting DSA-specific IgM and IgG-MBC differentiation using *in vitro* assay systems as an early diagnostic method and biomarkers that enable the inhibition of AMR development with less invasive therapeutic intervention (207). The findings supported the clinical applicability of the detection of DSA-specific IgM-MBC differentiation after elucidating the optimal conditions for inducing the differentiation of DSA-specific IgM-MBC differentiation into IgG-PCs, the detailed mechanism leading to AMR.

Alternatively, IVig has been administered for AMR control mainly, but IgM-enriched IVig has been reported to improve the pathological condition and prognosis in severe infectious diseases and organ ischemia (42, 253–256). Therefore, further elucidation of how signal mediated by IgM-enriched-IVig are involved in the AMR development may contribute to the establishment of AMR control.

Therefore, focusing on the involvement mechanism of humoral immunity-associated components in the pathological conditions regardless of the difference in fields and conventional knowledge and elucidating the mechanism by which these humoral immunity-associated components participate in AMR development will likely be applicable to the development of new diagnostic and therapeutic methods for improving AMR management.

## Author Contributions 

YM designed and wrote the paper. TW revised the paper. TW and X-KL provided excellent advice. All authors contributed to the article and approved the submitted version.

## Conflict of Interest

The authors declare that the research was conducted in the absence of any commercial or financial relationships that could be construed as a potential conflict of interest.
